# Instant Detection of Cerebral Blood Flow Changes in Infants with Congenital Heart Disease during Transcatheter Interventions

**DOI:** 10.3390/jcm13113115

**Published:** 2024-05-26

**Authors:** Martin Leth-Olsen, Gaute Døhlen, Hans Torp, Siri Ann Nyrnes

**Affiliations:** 1 Department of Circulation and Medical Imaging (ISB), Faculty of Medicine and Health Sciences, NTNU—Norwegian University of Science and Technology, 7030 Trondheim, Norwaysiri.a.nyrnes@ntnu.no (S.A.N.); 2 Children’s Clinic, St Olav’s University Hospital, 7030 Trondheim, Norway; 3 Department of Pediatric Cardiology, Oslo University Hospital, 0372 Oslo, Norway

**Keywords:** cerebral blood flow velocity, congenital heart disease, Doppler, transcatheter intervention, neuroprotection, transfontanellar Doppler

## Abstract

**Background**: Transcatheter interventions are increasingly used in children with congenital heart disease. However, these interventions can affect cardiac output and cerebral circulation. In this pilot study, we aimed to investigate the use of NeoDoppler, a continuous transfontanellar cerebral Doppler monitoring system, to evaluate the impact of transcatheter interventions on cerebral circulation. **Methods**: Nineteen participants under one year of age (mean age 3.5 months) undergoing transcatheter cardiac interventions were prospectively included. Transfontanellar cerebral Doppler monitoring with the NeoDoppler system was initiated after intubation and continued until the end of the procedure. **Results**: Instant detection of changes in cerebral blood flow were observed across a spectrum of transcatheter interventions. Balloon aortic valvuloplasty demonstrated temporary cessation of cerebral blood flow during balloon inflation. Increase in cerebral diastolic blood flow velocity and decreased pulsatility were observed during patent ductus arteriosus occlusion. Changes in cerebral blood flow patterns were detected in two patients who encountered complications during their transcatheter interventions. There was no significant change in Doppler parameters before and after the interventions for the entire patient group. High quality recordings were achieved in 87.3% of the monitoring period. **Conclusions**: Continuous transfontanellar cerebral Doppler is feasible in monitoring cerebral hemodynamic trends and shows instantaneous changes associated with interventions and complications. It could become a useful monitoring tool during transcatheter interventions in infants.

## 1. Introduction

Cardiac catheterization in children with congenital heart disease (CHD) has evolved from being a purely diagnostic field to becoming more treatment oriented [1]. Transcatheter interventions, which are minimally invasive and considered safe, have become the treatment of choice for selected patients with patent ductus arteriosus, secundum atrial septal defects, and pulmonary valve stenosis. These procedures have demonstrated few complications and low reintervention rates [2,3,4,5].

An increasing number of CHDs are now being treated by minimally invasive interventions with transcatheter balloon valvuloplasty/angioplasty, device closures, and stents as definitive treatment or part of hybrid approaches [1,6,7]. However, it is important to note that transcatheter interventions may affect cardiac output and cerebral circulation.

Transcranial Doppler can be used to monitor cerebral blood flow velocities (CBFVs) during transcatheter interventions and has shown clinically significant changes in brain perfusion during certain procedures [8,9,10,11]. However, these studies are now more than two decades old and cardiac interventions have evolved considerably since then. NeoDoppler is a new transfontanellar cerebral Doppler monitor for neonates and infants [12,13]. This technology provides continuous CBFV monitoring and has previously demonstrated its ability to detect high-intensity transient signals that represent micro-emboli in this patient group [14].

In this study we wanted to explore CBFVs and their dynamics during different transcatheter interventions in infants with CHD. We hypothesized that continuous transfontanellar cerebral Doppler monitoring can display the instant hemodynamic effects of procedures and detect early warning signs of complications.

## 2. Materials and Methods

### 2.1. Participants and Ethics

The trial was carried out at the Department of Pediatric Cardiology, Oslo University Hospital (OUS), Oslo, Norway. This was done in collaboration with the ultrasound group at the Department of Circulation and Medical Imaging, The Norwegian University of Science and Technology (NTNU), Trondheim, Norway. Participants were recruited at OUS from February 2019 to July 2020. A convenience sample of neonates and infants with CHD under one year of age, with an open fontanelle, who were scheduled for transcatheter cardiac interventions were eligible for inclusion. Neonates and infants with a small or closed fontanelle or thick hair, where an ultrasound signal was not possible to obtain, were excluded. The study was approved by the Regional Committee for Medical and Health Research Ethics, REC Central (Reference 2017/314), the Norwegian Directorate of Health and the Norwegian Medicines Agency (Reference 19/05458). Nineteen participants were included after informed and written consent was given from the parents of the participants.

### 2.2. The Transfontanellar Cerebral Doppler Monitoring System

NeoDoppler is a non-invasive ultrasound Doppler system developed by the ultrasound group at NTNU, Trondheim, Norway. The prototype system used in this study consists of a scanner (Manus EIM-A, Aurotech Ultrasound AS, Tydal, Norway), a small ultrasound probe (Imasonic SAS, Besançon, France), operating at 7.8 MHz with plane wave transmissions covering a cylindrical shape with a diameter of 10 mm and depth down to 38 mm. The system is connected to a PC with an in-house MATLAB R2021B (The MathWorks, Natick, MA, USA) application and displays real-time high-frame-rate color M-mode Doppler and Doppler spectrogram simultaneously. The in-house MATLAB application allows for adjustment of depth, sample volume, gain, vertical scale, low pass filter, and horizontal sweep during the recordings and in post processing [13]. The application was used for post processing and analysis of the Doppler recordings. Automatic Doppler tracings with calculation of peak systolic velocity (PSV), end diastolic velocity (EDV), time-averaged maximum velocity (TAV), pulsatile index (PI = (PSV − EDV)/TAV), and resistive index (RI = (PSV − EDV)/PSV) are generated based on the tracings. The sample volume was set to 5 mm and the depth chosen for the Doppler readings remained unchanged for each procedure, meaning the same artery was monitored consistently.

### 2.3. Monitoring Protocol

Transfontanellar cerebral Doppler monitoring with the NeoDoppler research system was initiated after intubation and the patients were monitored until the transcatheter procedure was completed. The probe was attached over the anterior fontanelle with a soft hat with a probe attachment mechanism as previously described [13]. During the entire procedure, Doppler recordings were continuously collected in intervals to allow for data storage and to adhere to the ALARA principle (“as low as reasonably achievable”). The transfontanellar cerebral Doppler monitor was blinded for all clinical personnel during the procedure; it was used only when attaching the probe to secure an adequate signal and for periodical signal quality checks. Clinical and procedural events were registered during the procedure in a time stamped document. Additional monitoring data from the electronic patient chart (Metavision 6.9, iMDsoft, Tel Aviv, Israel) were retrospectively collected (SpO2, FiO2, EtCO2, blood pressure, medications).

### 2.4. Feasibility and Quality Evaluation

The entire recording of each patient was evaluated visually and classified as follows: 1—the recording was of good quality with good automatic Doppler tracing; 2—automated tracing was not feasible, but manual inspection allowed for interpretation of the Doppler pattern; 3—not acceptable quality. An automatic quality metric (0–100%) was also calculated for each heartbeat, based on the correlation between consecutive heartbeats as described previously [13]. Only quality > 80% was considered as valid/high quality data. Valid fraction was defined as the time with valid data in percent of the total recording time.

### 2.5. Analysis of Doppler Measurements and Trends

Trend windows displaying the entire Doppler monitoring periods and simultaneous data from other monitoring equipment were generated for all patients and visually evaluated.

**Figure 1:**
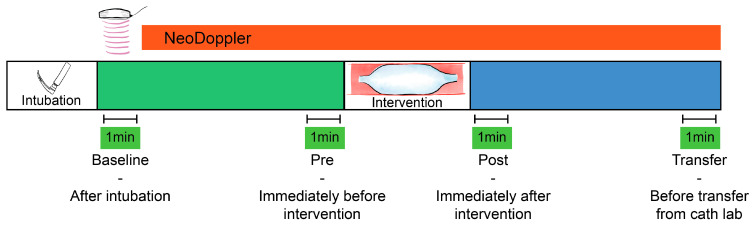
Monitoring cerebral blood flow velocities during transcatheter interventions. Four one-minute averages of cerebral blood flow velocities and indices were registered for statistical comparison. Baseline—after intubation, Pre—immediately before the intervention (e.g., balloon valvuloplasty), Post—immediately after the intervention, and Transfer—before transfer from the catheter laboratory.

A baseline measurement (Baseline, Figure 1) was calculated at the initiation of monitoring (directly after intubation), as a one-minute average of Doppler velocity measurements, Doppler indices, and available data from the other monitoring equipment. The baseline variables were compared to a one-minute average of identical parameters obtained after the procedure directly before transfer from the catheterization laboratory (Transfer, Figure 1). Percentage change from baseline measurements was calculated for all individual patients and the correlation of changes between CBFV and blood pressure were evaluated.

### 2.6. Interventions and Events/Complications

The immediate effect of transcatheter interventional procedures such as balloon valvuloplasty, device occlusions and other hemodynamic events were visually described based on the Doppler tracings and trend curves. Doppler parameters immediately before (Pre) and after (Post) the device interventions were also described, with one-minute averages for numeric comparison (Figure 1). If several procedural events were registered, the one we found to be most significant was selected for further numeric analysis. If several consecutive interventions were performed, the one-minute average Doppler tracing was calculated after the final intervention.

### 2.7. Statistical Analysis

Categorical variables are presented as count (percent). Continuous variables are presented as mean ± standard deviation for normally distributed data. The normality assumption was assessed by visual inspection of Q-Q plots. To evaluate variables before and after the transcatheter procedure, the differences were tested for normal distribution and a paired Student’s *t* test was performed; a *p*-value < 0.05 was considered statistically significant. The correlation between CBFV and blood pressure was evaluated with the Pearsson’s linear correlation coefficient; r > 0.5 was considered as a strong linear correlation and a *p* < 0.05 was considered significant.

### 2.8. Safety

The safety of this transfontanellar cerebral Doppler monitoring system was previously elaborated [13]. The temperature increase is highest at the skin surface; due to the unfocused beam, the temperature diminishes with increasing depth. Thermal and mechanical indices were continuously displayed on the display unit. The skin where the probe was attached was inspected after removal to assess for local adverse effects.

## 3. Results

### 3.1. Participants, Feasibility and Quality Evaluation

Informed consent was obtained from the parents of 19 participants. Three patients (15.8%) were excluded from the analysis because an adequate Doppler signal could not be obtained: One patient was excluded due to technical challenges with the research setup, one patient (seven months old) had a fontanelle that was too small to allow for high quality monitoring, and one patient (six months old) had hair that was too thick for feasible Doppler monitoring. No hair was removed to allow monitoring. Of the remaining 16 patients, fourteen patients (73.7%) had visually good quality recordings with good automatic tracing, one patient (5.25%) had only periods of good automatic tracing, and one patient (5.25%) had acceptable quality for visual evaluation but not for automatic tracing. The valid fraction for the included participants was 87.3%. Basic patient characteristics for the 16 patients (84.2%) included in the analyses are summarized in Table 1.

### 3.2. Trends

**Figure 2:**
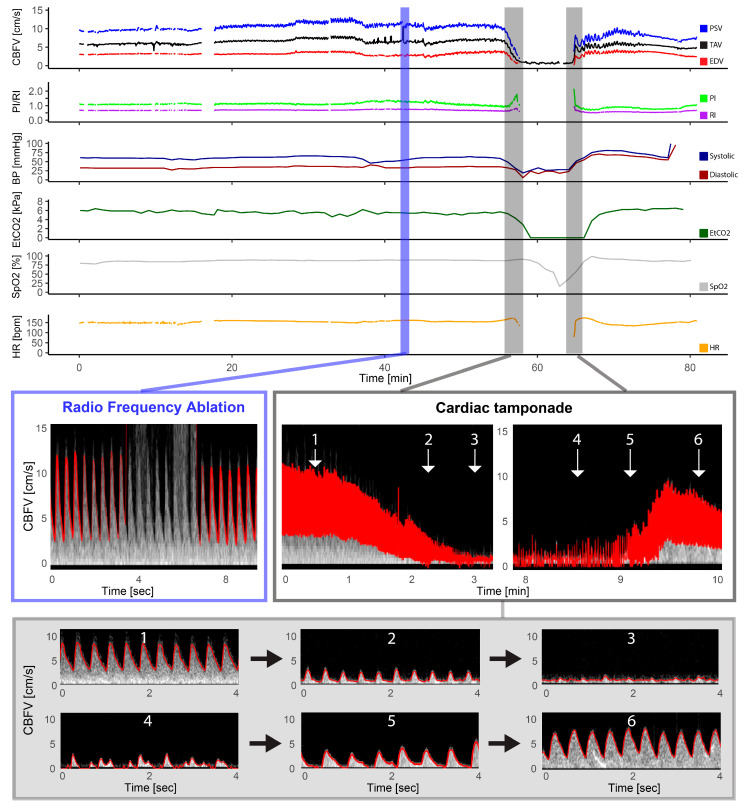
Pulmonary atresia with intact ventricular septum—radio frequency ablation. The figure illustrates the full monitoring period in a patient with pulmonary atresia with intact ventricular septum who underwent radio frequency ablation and had a complication in the form of a cardiac tamponade. The light blue section illustrates the artifact that the radio frequency ablation had on the Doppler signal with gray intense signal interfering with the automatic trace shown in red, but the Doppler signal being still visible. The section marked in gray represents the Doppler spectrograms obtained during the cardiac tamponade event, providing a visual representation of the hemodynamic changes that occurred: 1: Cerebral blood flow velocities (CBFV) before, 2: reduced CBFV during tamponade development, 3: severely reduced to absent CBFV during tamponade, 4: irregular peaks of CBFV, 5: increased CBFV (still reduced and absent end diastolic flow), and 6: CBFV after successful pericardiocentesis; also notice the high-intensity transient signal in the spectrum representing a microembolus. CBFV: Cerebral blood flow velocities, EDV: end-diastolic velocity, EtCO2: end-tidal CO2, HR: heart rate, PI: pulsatile index, PSV: peak systolic velocity, RI: resistive index, SpO2: pulsoxymetri oxygen saturation, TAV: time-averaged maximum velocity.

A selected example of an entire monitoring period is demonstrated in Figure 2. For the entire group, comparing Baseline (Figure 1) to measurements before Transfer (Figure 1) from the catheterization laboratory showed no significant change in cerebral blood flow velocities, Doppler indices, blood pressure, EtCO2 or heart rate after the transcatheter procedure. Table 2 summarizes changes in Doppler measurements and EtCO2 for all patients where Doppler quality was considered good in these selected timepoints (n = 15). There was a strong positive linear relationship between the change in EDV and diastolic blood pressure (r = 0.842, *p* < 0.001) but no significant relationship between PSV and systolic blood pressure (r = −1.125, *p* > 0.05). There was no significant linear relationship between the relative change in EtCO2 and cerebral blood flow velocities.

### 3.3. Interventions and Events

Changes in Doppler measurements one minute before and after interventions (pre and post) in all patients are summarized in Table 2. There was no significant change in CBFV measurements, CBFV indices, blood pressure, EtCO2, or heart rate in the period immediately before the intervention (Pre) compared to the period immediately after the intervention (Post) for the whole group.

### 3.4. Balloon Valvuloplasty/Angioplasty/Procedures

**Figure 3:**
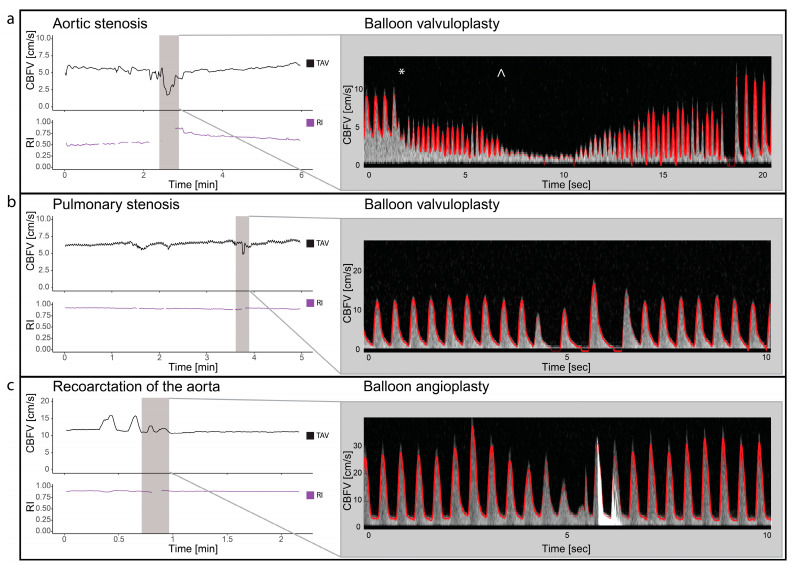
Balloon valvuloplasty/angioplasty. The figure illustrates cerebral blood flow velocities (CBFVs) and resistive index (RI) for three different selected examples of balloon valvuloplasty/angioplasty. It displays trends of time-averaged maximum velocity (TAV) and PI around the time of intervention and Doppler spectrograms with tracing (red) during the interventions. Panel (**a**) displays balloon dilatation of a valvular aortic stenosis. The start of ventricular pacing (*) with immediate reduced CBFV and increase heart rate followed by balloon dilatation (^) with significant drop in CBFV. Panel (**b**) displays the changes in CBFV in balloon valvuloplasty of valvular pulmonary stenosis. Two heart beats with reduced CBFV are seen, before 2 heart beats with increased CBFV but no end diastolic flow is seen. Panel (**c**) illustrates the changes in CBFV in a case with coarctation of the aorta during balloon angioplasty. Notice the initial increase, before a reduction in CBFV before it returns to baseline. The high-intensity transient signal in the spectrum is the effect of balloon rupture. CBFV: cerebral blood flow velocities, RI: resistive index, TAV: time-averaged maximum velocity.

**Figure 4:**
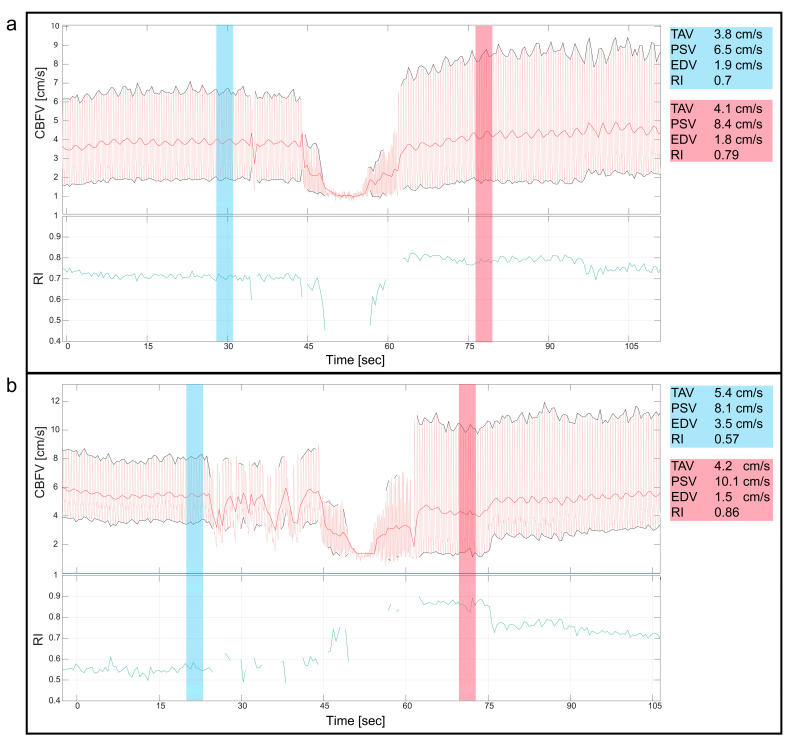
Valvular aortic stenosis, balloon valvuloplasty. This figure shows the cerebral blood flow velocity (CBFV) traces and resistive index (RI) during two cases of balloon valvuloplasty of valvular aortic stenosis in neonates. Both figures (**a**,**b**) show the immediate reduction in CBFV during the procedure, where the initial decline in CBFV is due to ventricular pacing before balloon inflation, reducing CBFV even more. Notice, in both panels, the changes of the CBFV before (blue) and after (red) the intervention with a clear increase in peak systolic velocities and increased pulsatility immediately after the intervention, also displayed with numeric values blue (before) and red (after). CBFV: cerebral blood flow velocity, EDV: end diastolic velocity, PSV: peak systolic velocity, RI: resistive index, TAV: time-averaged maximum velocity.

**Figure 5:**
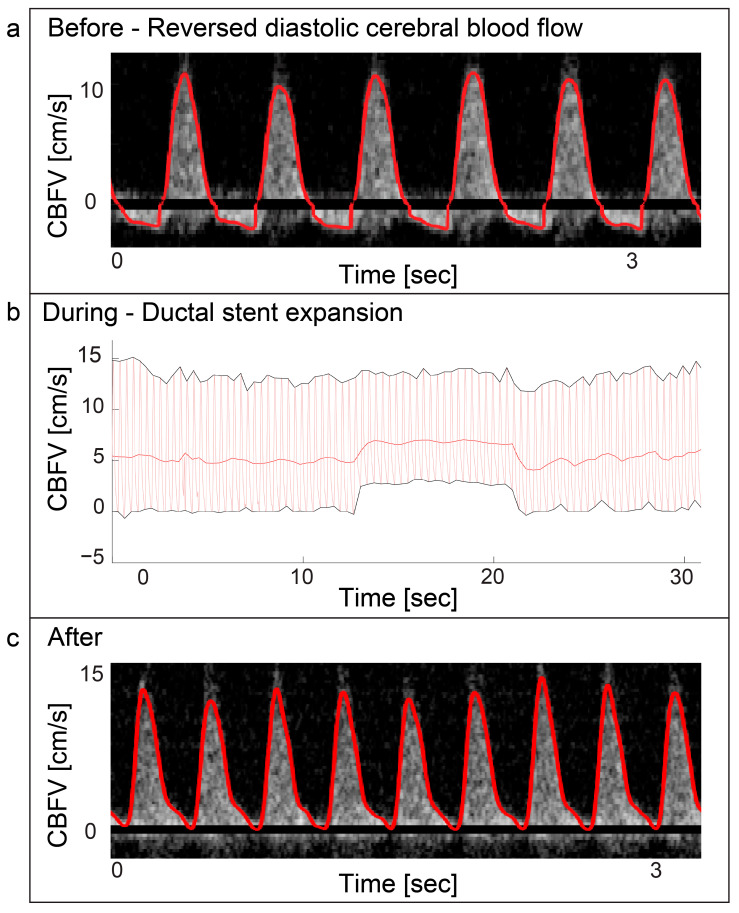
Ebstein anomaly—ductal stenting. Panel (**a**) displays a Doppler spectrogram with tracing (red) showing diastolic reversal of cerebral blood flow. This was periodically seen prior to ductal stenting in a patient with duct dependent Ebstein anomaly. The patient received prostaglandin E1 infusion prior to the procedure. Panel (**b**) displays the immediate increase of cerebral end-diastolic velocity (EDV) during ductal stent expansion with balloon inflation and return to baseline after balloon deflation in the same patient. Panel (**c**) displays the Doppler spectrogram with tracing (red) of the cerebral blood flow velocities after the procedure was performed. The cerebral blood flow velocity still displays high pulsatility with EDV approaching zero, but no reversal is seen. CBFV: Cerebral blood flow velocity.

Two patients with valvular aortic stenosis underwent aortic valvuloplasty. The immediate effects of ventricle pacing, balloon inflation and deflation on cerebral blood flow are seen in Figure 3 and Figure 4. Additionally, signs of increased peak velocities and RI immediately after balloon deflation and the stop of pacing were demonstrated. During the balloon valvuloplasty procedure for pulmonary stenosis, a few heartbeats exhibited reduced velocity, coinciding with the duration of balloon dilation (Figure 3). Additionally, a variable peak-to-peak time interval, which could be attributed to the extrasystoles associated with the procedure, was observed. For aortic coarctations, only short-lasting changes were seen during balloon inflation; Figure 3 shows an example of immediate effects of inflation on CBFV. The Doppler output in a patient with Ebstein anomaly is illustrated in Figure 5, demonstrating the immediate and transient effects of ductal stenting. Upon inflation of the balloon, there is an immediate increase in EDV and a simultaneous reduction in RI, attributable to decreased ductal shunting. This change is instantly displayed. Notably, this patient received prostaglandin E1 prior to stenting and exhibited periods of reversed EDV prior to stent placement. This reversal was not seen after the stent was placed (Figure 5).

### 3.5. Patent Ductus Arteriosus

**Figure 6:**
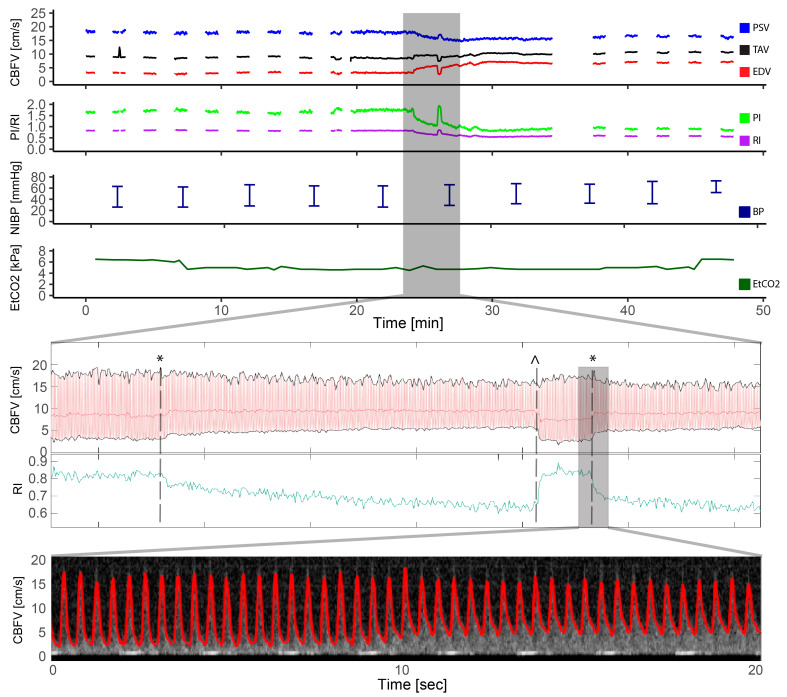
Patent ductus arteriosus. This figure illustrates the entire course of a transcatheter patent ductus arteriosus closure in a patient with a 6 mm patent ductus arteriosus. The cerebral blood flow velocity tracings and resistive index are highlighted in the period of device placement and illustrates the moment of ductal occlusion with an instant increase in end diastolic velocity (EDV) and decreased resistive index (RI). The device had to be repositioned with corresponding decrease in EDV and increase of RI at the moment of device removal, before the device once again was opened, and the ductus was closed with EDV increase and RI decrease. The two moments of ducal occlusions are illustrated with * and the moment of device removal with ^. The Doppler spectrogram with tracing in red is highlighted during the second ductus occlusion where the instant increase in EDV is seen. CBFV: cerebral blood flow velocity, EDV: end-diastolic velocity, EtCO2: end-tidal CO2, HR: heart rate, PI: pulsatile index, PSV: peak systolic velocity, RI: resistive index, SpO2: pulsoxymetri oxygen saturation, TAV: time-averaged maximum velocity.

PDA occlusion was performed in two patients. One patient had a 6 mm PDA closed with a Amplatzer vascular plug II, 10 mm. This patient had a 118% increase in EDV, 47% reduction in PI, and 32% reduction in RI after the procedure, compared to baseline. Visual inspection of the Doppler spectrogram and velocity tracings demonstrated immediate changes in EDV and pulsatility the moment the device was deployed (Figure 6), readjusted, repositioned, and finally occluded. For the second patient who underwent PDA closure, a 3 mm duct was occluded with an Amplatzer duct occluder where a 26% increase in EDV, compared to baseline, was seen after the procedure. PI was reduced by 27% and RI by 17%. Inspection of the Doppler patterns also demonstrated an immediate effect on EDV and pulsatility. The latter patient had device embolization after the procedure and was taken to the catheterization laboratory for snaring of the device and reocclusion on the second day. The Doppler pattern before snaring and reocclusion revealed increased pulsatility: PI from 0.8 to 1.46 (166%) and RI from 0.58 to 0.78 (134%) compared to the previous day. Snaring and successful reocclusion was performed with an immediate drop in flow pulsatility (PI 0.98 and RI 0.63 at the end of the procedure).

### 3.6. Pulmonary Atresia

One patient with pulmonary atresia had complications with a cardiac tamponade during radio frequency ablation of the pulmonary valve (Figure 1). The gradual decline in cerebral Doppler velocities was evident during the period of tamponade development. After successful resuscitation and pericardiocentesis, the increase in CBFV was demonstrated in the Doppler trend curves. The patient received three boluses of epinephrine (30 mcg) and a distinct increase in EDV was seen after reperfusion together with decreasing pulsatility.

## 4. Discussion

This study demonstrates that continuous transfontanellar cerebral Doppler can monitor hemodynamic trends and detect immediate changes in CBFV during transcatheter cardiac interventions in neonates and infants with CHD. This type of monitoring can also provide early warning signs of complications. This is clinically important information for the interventionist and anesthesiologist treating the patient.

### 4.1. Monitoring Trends and Events

There was no significant change in hemodynamic parameters from baseline to after the procedure for the full patient group. However, analyzing the whole group in one does not illustrate the important heterogeneity of the data and the need for personalized medicine. In some patients, a clear change in Doppler parameters is seen from baseline to after the intervention, which can be expected from the nature of the procedure and the desired change in hemodynamics. This is exemplified with the two inclusions with PDA where there is an increase in EDV and reduced pulsatility after intervention. This has previously been shown with transcranial Doppler in infants during transcatheter intervention and in surgical ligations of PDA [10,11,15,16]. In this material, there was one patient with a complication in the form of embolization of the ductus device after the initial occlusion. Subsequent measurement with NeoDoppler the day after the initial PDA closure showed increased pulsatility. The patient was not monitored with cerebral Doppler after transfer from the catheterization laboratory, but one could speculate that bedside monitoring with cerebral Doppler could have detected the changes in pulsatility associated with embolization/reopening of the shunt. It would be interesting to see if this technology could be used as a bedside monitoring tool to evaluate patients with significant shunts after interventional of medical PDA closure, potentially reducing the need of advanced echocardiography and radiation associated with X-ray. Furthermore, the potential use in premature infants with PDA is intriguing. Previous studies found cerebral ultrasound Doppler useful in the evaluation of PDA in prematures [17,18]. PDA in premature infants is associated with morbidity and mortality but the prevention and treatment of the open duct is still a controversial and challenging topic [19]. The hemodynamic understanding of the transitional period of premature infants needs to be explored further.

### 4.2. Monitoring to Detect Complications

In this study, two patients had complications due to the interventions. These complications, namely tamponade and device embolization during PDA closure, were seen as distinct and immediate changes in Doppler parameters. In the cardiac tamponade, invasive systemic blood pressure curves were available; however, this is not always the case in transcatheter interventions. Transfontanellar CBFV monitoring could serve as a valuable tool for detecting complications by monitoring cardiac output and cerebral blood flow. The immediate changes in Doppler velocities observed in this study underscore the potential utility of this approach in identifying complications and undesired effects. Furthermore, the immediate changes that were observed suggest that interventionists have the possibility to monitor the direct effects of interventions on cerebral blood flow velocities as they occur in real-time.

Direct cerebral monitoring during cardiac transcatheter interventions in children.

Transcranial Doppler devices have been utilized in studies involving children with CHD but are not used regularly in the clinical setting [8,9,10,11]. Furthermore, the attachment mechanisms that these devices employ are not appropriate for infants. Near-infrared spectroscopy (NIRS) is used more frequently in children with congenital heart disease, especially during surgery and in the postoperative period [20]. The institution where this study was performed does not use NIRS routinely during transcatheter interventions. NIRS has been studied for its potential in monitoring cerebral oxygenation during different transcatheter procedures and in predicting complications, but more evidence is needed to show clinical benefits [21,22,23]. A previous study demonstrated that NIRS has a delayed response compared to CBFV in acute changes in cerebral perfusion during cardiac surgery with cardiopulmonary bypass in infants [24]. Invasive and non-invasive blood pressure monitoring are routinely used as a surrogate for cardiac output and cerebral perfusion during interventions. However, not all transcatheter procedures require invasive continuous intraarterial blood pressure and rely on periodic non-invasive pressure monitoring. In this study only three patients had continuous invasive arterial blood pressure monitoring. In the cases without continuous invasive blood pressure monitoring, a non-invasive alternative with continuous data and immediate feedback could be especially useful, i.e., illustrated by the example with the tamponade in this study.

We also demonstrated an example of cerebral reversal of diastolic flow in the case of duct-dependent circulation. Diastolic reversal could be indicative of too large a shunt with pulmonary hyperperfusion with systemic and cerebral steal. A recent article by Mir et al. showed steal in the cerebral circulation of newborns with CHD in the first week of life, before surgery [25]. These findings suggest that the maintenance of ductal patency could potentially influence cerebral perfusion. The evaluation of systemic to pulmonary shunts with cerebral Doppler was previously proposed for optimization of surgically placed shunts [26,27]. The ease of use of continuous transfontanellar Doppler in this patient group makes it optimal in evaluating and optimizing shunts to balance pulmonary and systemic perfusion.

### 4.3. Future Research

The wide range of diagnosis, procedures, and even age range of the neonates and infants in this study displays multiple possible use-case scenarios of this technology. Hypotheses generated based on this pilot study can be the basis for future research, especially PDA patients, which is a group that would be valuable to study both for catheter interventions and also during medical intervention in the premature.

### 4.4. Limitations

A limitation of this study is the small sample size and the heterogenicity of the participants. Evaluation of the cerebral hemodynamic effect of the different interventions and diagnosis requires a more uniform group of patients.

In this study it was not possible to record synchronized electrocardiogram signals. It would be an advantage to have an electrocardiogram in the analysis, as arrhythmias and extrasystoles are common during interventions and cause changes in Doppler velocities. During balloon valvuloplasty of pulmonary valves, the effect of occlusion and the effect of triggered extrasystoles in the Doppler signal are difficult to separate without an electrocardiogram. However, extrasystoles can be suspected based on the change in peak-to-peak intervals in the Doppler signal.

Furthermore, an important limitation is the lack of neurocognitive follow-up or cerebral magnetic resonance imaging of the patients in this study. However, this was beyond the scope of this pilot study.

## 5. Conclusions

Continuous transfontanellar cerebral Doppler monitoring is feasible in neonates and infants during cardiac transcatheter interventions. This monitoring adds insights into the trends of CBFV and can provide immediate feedback to clinicians during procedures. Instantaneous changes associated with hemodynamically significant interventions or complications can be displayed. Continuous transfontanellar cerebral Doppler monitoring could become a useful monitoring tool for the interventionist and the anesthesiologist during transcatheter interventions in the future.

## Figures and Tables

**Table 1:** Basic clinical data. Categorical variables are presented as count (percent). Continuous variables are presented as mean ± standard deviation.

Basic Clinical Data (Units)	
Age (months)	3.5 ± 2.8
Weight (g)	5187 ± 1977
BSA (m^2^)	0.29 ± 0.08
Female (*n*)	6 (37.5%)
Blood pressure systolic (mmHg)	85 ± 13
Blood pressure diastolic (mmHg)	49 ± 9
Mean blood pressure (mmHg)	63 ± 9
HR (bpm)	134 ± 24
Cerebral Doppler monitoring time (min)	72.8 ± 38.2

**Table 2:** Monitoring trends. One-minute averaged measurements of time-averaged maximum velocities (TAV), resistive index (RI), and end tidal CO2 from baseline (after intubation immediately after establishing Doppler monitoring), pre (the minute before the interventional procedure), post (the minute immediately after the interventional procedure), and transfer (before transfer from the catheterization laboratory). Measurements are presented as absolute values and as percentages compared to baseline for all individual patients.

		TAV cm/s (%)				RI (%)				EtCO2 kPa (%)			
Diagnosis	Procedure	Baseline	Pre	Post	Transfer	Baseline	Pre	Post	Transfer	Baseline	Pre	Post	Transfer
vPS	Balloon valvulopasty	7.28 (100%)	6.65 (91%)	6.45 (89%)	6.67 (92%)	0.78 (100%)	0.9 (115%)	0.91 (117%)	0.9 (115%)	4.2 (100%)	3.2 (76%)	3 (71%)	3.3 (79%)
reCoA	Balloon angiopasty	13.61 (100%)	13.97 (103%)	13.59 (100%)	13.84 (102%)	0.79 (100%)	0.76 (96%)	0.76 (96%)	0.75 (95%)	5.1 (100%)	5.2 (102%)	5.1 (100%)	5.5 (108%)
vPS	Balloon valvulopasty	6.95 (100%)	4.79 (69%)	5.06 (73%)	4.81 (69%)	0.82 (100%)	0.81 (99%)	0.8 (98%)	0.76 (93%)	5.85 (100%)		4.7 (80%)	4.5 (77%)
HLHS	Sano shunt test occlusion	8.68 (100%)	9.15 (105%)	8.98 (103%)	9.12 (105%)	0.78 (100%)	0.76 (97%)	0.72 (92%)	0.78 (100%)				
vPS	Balloon valvulopasty	8.42 (100%)	8.39 (100%)	7.32 (87%)	7.92 (94%)	0.66 (100%)	0.73 (111%)	0.74 (112%)	0.74 (112%)	5.35 (100%)	5.7 (107%)	5.6 (105%)	5.4 (101%)
svPS	Balloon valvulopasty	10.75 (100%)	10.93 (102%)	11.86 (110%)	11.89 (111%)	0.72 (100%)	0.76 (106%)	0.73 (101%)	0.74 (103%)	4.9 (100%)	4.6 (94%)	4.9 (100%)	5.2 (106%)
reCoA	Balloon angiopasty	14.91 (100%)	12.03 (81%)	11.15 (75%)	11.79 (79%)	0.81 (100%)	0.89 (110%)	0.89 (110%)	0.87 (107%)	6.7 (100%)	6.2 (93%)	6.1 (91%)	6.15 (92%)
Ebstein anomaly	PDA stent	3.84 (100%)	5.79 (151%)	5.26 (137%)	5.01 (130%)	0.99 (100%)	0.95 (96%)	1 (101%)	0.99 (100%)	5.03 (100%)	5.3 (105%)	5.3 (105%)	5.15 (102%)
PDA	Occlusion	9.41 (100%)	8.45 (90%)	10.34 (110%)	10.68 (113%)	0.82 (100%)	0.82 (100%)	0.55 (67%)	0.56 (68%)	6.43 (100%)	5 (78%)	5 (78%)	6.45 (100%)
vAS	Balloon valvulopasty	4.34 (100%)	3.58 (82%)	4.29 (99%)	4.28 (99%)	0.56 (100%)	0.73 (130%)	0.77 (138%)	0.7 (125%)	4.4 (100%)	4.3 (98%)	4.3 (98%)	4.5 (102%)
vAS	Balloon valvulopasty	5.58 (100%)	5.21 (93%)	5.04 (90%)	5.52 (99%)	0.53 (100%)	0.55 (104%)	0.76 (143%)	0.59 (111%)	5.35 (100%)	5.4 (101%)	5.2 (97%)	4.75 (89%)
PA-IVS	Radio frequency ablation	5.95 (100%)	6.99 (117%)	4.35 (73%)	5.61 (94%)	0.71 (100%)	0.65 (92%)	0.61 (86%)	0.53 (75%)	5.4 (100%)	5.4 (100%)		6.4 (119%)
LPA stenosis	Balloon valvulopasty	13.56 (100%)	12.87 (95%)	13.64 (101%)	13.56 (100%)	0.76 (100%)	0.78 (103%)	0.78 (103%)	0.79 (104%)	4.85 (100%)	5.05 (104%)	4.8 (99%)	4.9 (101%)
ASD secundum	Occlusion	9.62 (100%)	9.25 (96%)	9.54 (99%)	8.76 (91%)	0.85 (100%)	0.88 (104%)	0.86 (101%)	0.87 (102%)				
PDA	Occlusion	23.27 (100%)	21.49 (92%)	22.7 (98%)	23.44 (101%)	0.7 (100%)	0.77 (110%)	0.71 (101%)	0.58 (83%)	6.6 (100%)	5.9 (89%)	6 (91%)	6.5 (98%)

ASD = atrial septal defect EtCO2 = end tidal CO2, HLHS = hypoplastic left heart syndrome, LPA = left pulmonary artery, PA-IVS = pulmonary atresia with intact ventricular septum, PDA = patent ductus arteriosus, reCoA = recoarctation of the aorta, RI = resistive index, svPS = supravalvular pulmonary stenosis, TAV = time-averaged maximum velocity, vAS = valvular aortic stenosis, vPS = valvular pulmonary stenosis.

## Data Availability

The datasets analyzed during the current study are available from the corresponding author on reasonable request. The data are not publicly available due to data privacy law.
